# Revisiting typhoid fever surveillance in low and middle income countries: lessons from systematic literature review of population-based longitudinal studies

**DOI:** 10.1186/s12879-016-1351-3

**Published:** 2016-01-29

**Authors:** Vittal Mogasale, Vijayalaxmi V. Mogasale, Enusa Ramani, Jung Seok Lee, Ju Yeon Park, Kang Sung Lee, Thomas F. Wierzba

**Affiliations:** 1 Policy and Economic Research Department, International Vaccine Institute, SNU Research Park, 1 Gwanak-ro, Gwanak–gu, Seoul 151-742 South Korea; 2 Epidemiology Department, International Vaccine Institute, Seoul, South Korea; 3 Biostatistics and Data Management Department, International Vaccine Institute, Seoul, South Korea; 4 Development and Delivery Unit, International Vaccine Institute, Seoul, South Korea; 5 PATH, 455 Massachusetts Avenue NW, Suite 1000 Washington, DC USA

**Keywords:** Typhoid fever, Surveillance, Systematic literature review, Low and middle income countries, Under-estimation

## Abstract

**Background:**

The control of typhoid fever being an important public health concern in low and middle income countries, improving typhoid surveillance will help in planning and implementing typhoid control activities such as deployment of new generation Vi conjugate typhoid vaccines.

**Methods:**

We conducted a systematic literature review of longitudinal population-based blood culture-confirmed typhoid fever studies from low and middle income countries published from 1^st^ January 1990 to 31^st^ December 2013. We quantitatively summarized typhoid fever incidence rates and qualitatively reviewed study methodology that could have influenced rate estimates. We used meta-analysis approach based on random effects model in summarizing the hospitalization rates.

**Results:**

Twenty-two papers presented longitudinal population-based and blood culture-confirmed typhoid fever incidence estimates from 20 distinct sites in low and middle income countries. The reported incidence and hospitalizations rates were heterogeneous as well as the study methodology across the sites. We elucidated how the incidence rates were underestimated in published studies. We summarized six categories of under-estimation biases observed in these studies and presented potential solutions.

**Conclusions:**

Published longitudinal typhoid fever studies in low and middle income countries are geographically clustered and the methodology employed has a potential for underestimation. Future studies should account for these limitations.

**Electronic supplementary material:**

The online version of this article (doi:10.1186/s12879-016-1351-3) contains supplementary material, which is available to authorized users.

## Background

Typhoid fever is a serious systemic illness transmitted through fecal-oral route and known to affect population having limited water and sanitation infrastructure [1]. Presence of long-term carrier status and variable level of risk factors such as contaminated water, food and poor sanitation conditions in different geographical regions often causes patchy outbreaks and uneven disease distributions [2]. The disease is considered an important public health problem and the World Health Organization (WHO) has recommended vaccination with existing Vi polysaccharide vaccine targeting high-risk areas where typhoid fever is a problem [1]. For targeted vaccination strategies, it is essential to have a robust surveillance to understand the distribution of typhoid fever by geography and population groups. Besides quantifying disease burden, surveillance is helpful in tracking changes in level of incidence, anti-microbial resistance and the impact of typhoid fever control interventions. The importance of surveillance has increased with imminent availability of new generation typhoid conjugate vaccines [3], which may necessitate revisiting of WHO policies on vaccination strategies.

It is challenging to measure the real disease burden of typhoid fever based on a surveillance system. First, typhoid fever is common in locations with poor water and sanitation, where infrastructure and resources required for good surveillance are limited. Sustaining long-term effective surveillance needs continued commitment of health system and scarce resources. Second, because typhoid fever often clinically resembles other febrile illnesses, it is clinically misdiagnosed in many regions of the world where malaria and dengue are highly prevalent [1]. In remote and resource poor settings without laboratory surveillance systems, typhoid fever outbreaks as well as routine cases are under reported [4]. Third, the current typhoid fever diagnostics have limitations in terms of availability and reliability which exemplifies the misrepresentation of the disease [2]. Typhoid fever is often diagnosed clinically or using antibody titers in routine settings at hospitals and health facilities. The diagnostic sensitivity and specificity of antibody titer based tests is less than optimal [5]. Blood culture is highly specific but its sensitivity is lower, ranging from 40 to 80 % [6], and poses several operational and laboratory challenges [5]. Forth, only a proportion of all febrile cases at community may choose to visit health care facilities where surveillance is conducted underrepresenting true number of cases at the community. This is because people with febrile illness may opt for different service providers such as alternative health facilities, private practitioners, pharmacies, traditional healers or self-medication or no treatment [7].

Despite these challenges, global donors, policy and financing bodies and local decision makers look for precise information on size and seriousness of typhoid fever problem. Therefore, it is critical to identify and list studies that tried to minimize surveillance challenges while measuring the incidence of typhoid fever. Such studies will help in pinpointing the geographical locations where typhoid continues to be an important problem so that interventions can be targeted. We conducted a systematic literature review to identify blood culture-confirmed typhoid fever studies that try to represent the disease burden in underlying community. Based on the literature review we summarized the current challenges and future surveillance needs for improving the precision of disease burden estimates.

## Method

We performed the systematic literature review and presented results as per the Preferred Reporting Items for Systematic Reviews and Meta-analyses (PRISMA) statement (please see additional file 1).

### Search strategy and selection criteria

We undertook a systematic literature review of population-based, longitudinal studies of blood culture-confirmed typhoid fever conducted in low and middle income countries [8] published from 1^st^ January 1990 to 31^st^ December 2013 using a predefined protocol. The search involved PubMed and Embase as primary electronic databases for identification of publications. Pan American Health Organisation (PAHO) and WHO websites were utilized to identify additional publications. The key words used were (“typhoid” OR “typhoid fever” OR “*Salmonella* Typhi” OR “*S*. Typhi” OR “salmonella infection” OR “enteric fever”) AND (“incidence” OR “rate” OR “frequency” OR “prevalence” OR “morbidity” OR “burden” OR “surveillance” OR “epidemiology”). The search was limited to English language and studies of human individuals. The detailed inclusion and exclusion criteria are given below (Table 1). The search was independently conducted by two researchers and the results were compared. Any differences between two researchers were resolved based on discussion and agreement, if unresolved, third independent researcher made the final decision. All selected papers were reviewed by a third researcher before data extraction to confirm its adherence to inclusion criteria and to limit the risk-of bias. In the final list, we included distinct population-based longitudinal studies that used blood culture for typhoid fever confirmation in incidence estimation irrespective of intensity of surveillance.

**Table 1:** Inclusion and exclusion criteria for systematic literature review

Inclusion Criteria
• Published from 1^st^ January, 1990 to 31^st^ December 2013
• Listed in Pubmed OR Embase OR WHO OR PAHO data base
• Conducted in low and middle income countries based on World Bank definition [8]
• Blood culture was used for typhoid fever confirmation
• Diagnostic health facility covers clearly defined population OR a health care utilization survey provides a population denominator
• Study conducted in human subjects
• Study published in English
Exclusion Criteria
• Surveillance that did not deploy blood culture for typhoid fever confirmation
• Results from intervention arm of clinical trials
• Typhoid fever outbreak reports
• Government reports based on selected sentinel health facilities
• Studies estimating incidence based on mathematical models
• Hospital based surveillance where denominator is not defined or health care utilization survey was not conducted

### Data analysis

We used both qualitative and quantitative and methods in data analysis. First, we qualitatively scrutinized study methodology that could have influenced incidence rate estimates. Based on the description presented in the paper, we identified the potential reasons for typhoid fever underestimation and determine the potential correction approaches. Then, we extracted data related to typhoid fever incidence and hospitalization rates from selected studies and calculated incidence correction factors for each study. Finally, the incidence rates were presented based on two estimates: a) without correcting limitations of surveillance methods, and b) after correcting the limitations of surveillance methods.

We did not include incidence rates from clinical trials because they did not present sufficient details on surveillance to correct for methodological limitations. The hospitalization rate was estimated based on the number of hospitalizations among confirmed typhoid fever cases during the surveillance which was not corrected for its methodological limitations. We used meta-analysis approach in summarizing the hospitalization rates, where weighted mean was estimated by regions using random effects model. Qualitative information such as methodology, challenges faced, strengths and limitations were descriptively summarized for considerations in future surveillance.

## Results

**Fig. 1:**
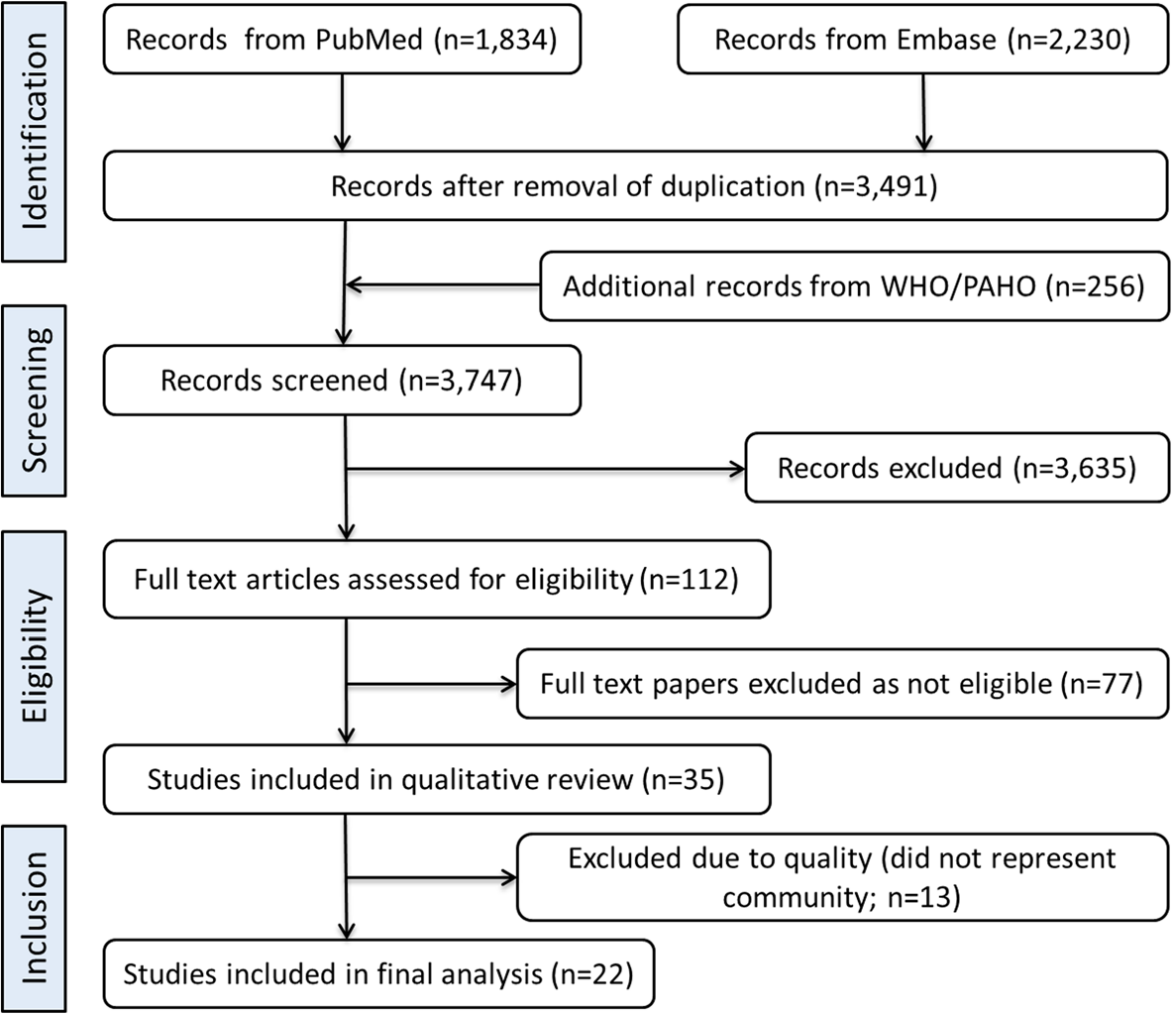
PRISMA diagram for systematic literature review conducted to identify population based longitudinal typhoid fever studies

Our systematic literature search resulted in 3747 English publications from 1990 to 2013 (Fig. 1). After reviewing titles and abstract 3635 irrelevant publications were excluded and full text articles for remaining 112 papers were obtained. Of them, 77 papers did not match inclusion criteria as they did not use blood culture for case confirmation and were excluded. Of 35 selected studies, 13 either did not represent a community or used modeling methods to estimate typhoid incidence. Finally we identified and analyzed data from 22 papers.

**Fig. 2:**
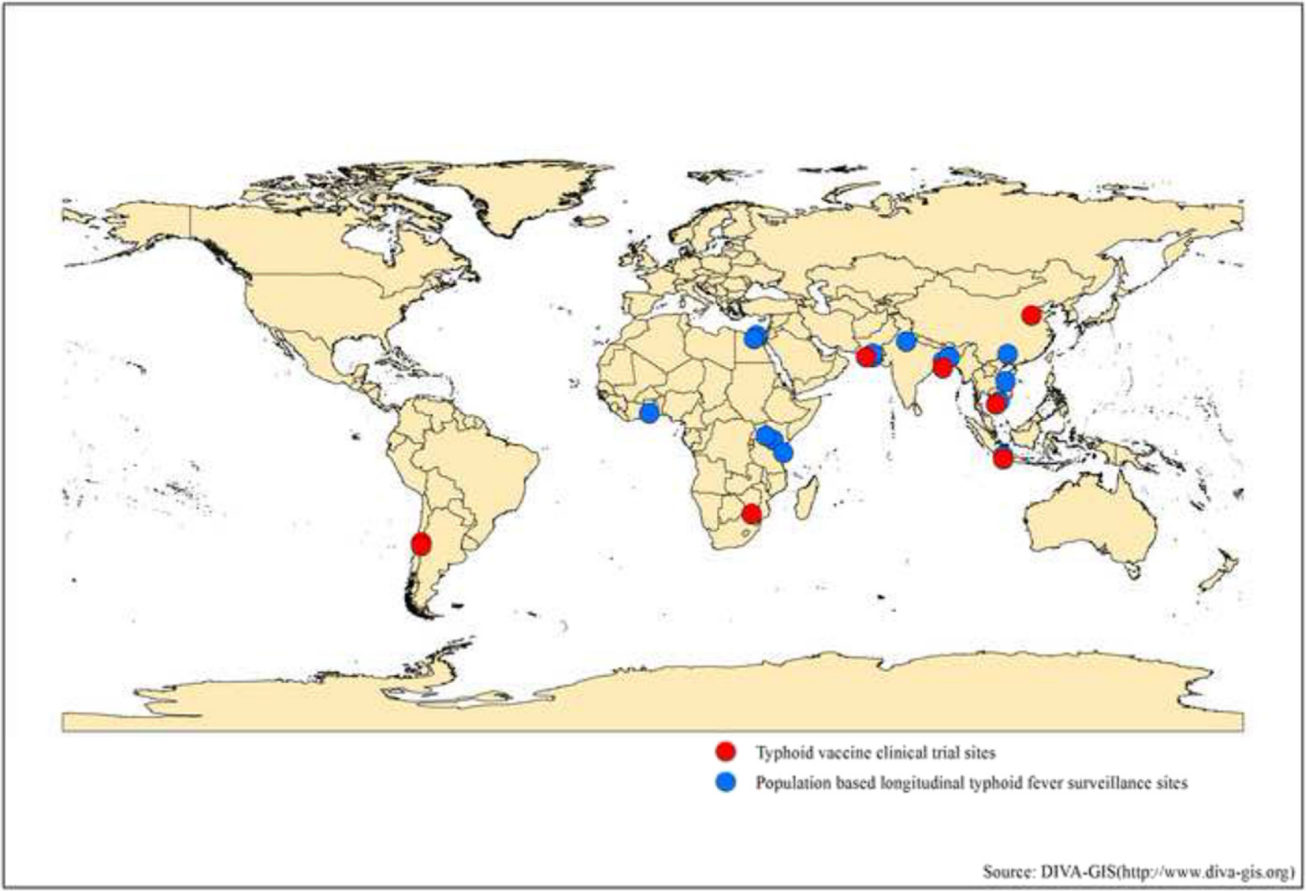
Geographical location of population- based longitudinal typhoid fever studies identified based on systematic literature review (Source: DIVA-GIS (http://www.diva-gis.org))

The 22 papers presented population-based blood culture-confirmed typhoid fever incidence estimates from 20 distinct sites in low and middle income countries [9–30] (Fig. 2). Based on United Nations region classification [31], most of published estimates were from Asia, with five estimates from the Southern Asia region [15, 17–21, 29, 30], four from the South-Eastern Asia region [15, 22–25], and two from the Eastern Asia region [15, 16]. The rest of the estimates were collected from Africa, with three sites in the Eastern Africa region [13, 14], two from Northern Africa [9, 10] and one from Western Africa [11, 12]. Except four estimates from Chile [27, 28], Southern Africa [26] and Indonesia [22], rest had incidence data collected post 1990s. Three papers from Pakistan [15, 19, 20] presented incidence data for different time period from same sites. Similarly, two papers each presented data for different periods from same sites in Ghana [11, 12], India [15, 29], Bangladesh [18, 30], Indonesia [15, 23] and Vietnam [24, 25].

Of the 22 studies, eight were of clinical trials restricted to certain age groups such as school children and young adults while excluding children below 2 years [16, 20, 22, 25–29]. These trials described pre-existing known high incidence typhoid fever locations. The papers focused on clinical trial related descriptions, surveillance related information was often sketchy without detailed methodological information and were excluded from detailed analysis.

The descriptions on surveillance below included 14 papers representing 15 distinct sites. This includes multi-site studies [13, 15] and, multiple studies from same sites in Ghana [11, 12], Bangladesh [18, 30] and Pakistan [15, 19] which used standardized methodologies (Table 2). Of the total 15 studies, seven (46 %) were located urban sites, four (27 %) in rural and remaining four (27 %) in mixed urban-rural sites (Table 2). The surveillance period ranged from November 1995 to December 2010 while the duration of individual study varied from 4 months to 36 months. The studies covered 4.0 million populations for 281 months and identified 63,220 eligible cases. Analysis included 41,325 subjects who provided blood samples and 1149 were found positive for *Salmonella* Typhi infection.

**Table 2:** Typhoid fever annual incidence rate in population-based, longitudinal studies published from 1^st^ January 1990 to 31^st^ December 2013 (not corrected for blood culture sensitivity)

Location	Year	Rural/urban	Duration (months)	Surveillance type	Inclusion criteria	Population covered by surveillance site^a^	Population utilizing the surveillance site	Eligible cases identified	Consented and provide blood sample	Included in final analysis	Surveillance method adjusted denominator^b^	Total blood culture- confirmed typhoid fever cases	Annual crude incidence/100,000	Surveillance method adjusted^b^ annual incidence/100,000	Source
Africa															
Belbeis district, Sharkia, Egypt	July 2001-October 2001	Rural + Urban	4	Passive sentinel sites (1 hospital + 11 fever specialists + 68 health providers + baseline census + health care utilization adjustment)	≥6mths of age; Current fever of ≥3 days	664,000	664,000	449	449	449	664,000	19	6^ac^	6^ac^	[9]
Fayoum, Egypt	June 2002-October 2002	Rural + Urban	5	Passive sentinel (1 hospital + 6 district hospitals + 16 infectious disease specialists + 13 rural health unit physicians + 18 primary care providers)	≥1 year age; Current fever of 38 °C for ≥2 days; OR clinically suspected typhoid fever	2,240,000	2,240,000	1815	1815	1804	766,540	90	10	29	[10]
Ashanti region, Ghana	September 2007-November 2008	Rural	13	Passive (1 hospital) + health care utilization adjustment	5-15 years age; Hospitalized; every second case	9600	9600	1456	1456	1456	4800	7	67	135	[11]
Ashanti region, Ghana	Sept 2007- July 2009	Rural	23	Passive (1 hospital) + health care utilization adjustment	<5 years age; hospitalized; every second case	22,425	5333	1351	1351	1196	2667	17	166	333	[11, 12]
Kibera, Kenya	March 2007- February 2009	Urban slum	24	Active (field clinic) + biweekly house to house visit + baseline census + health care utilization adjustment	All age; Current fever of 38 °C; OR respiratory illness^d^	28,000	54,535^e^	7852	1531	1531	16,423^e^	135	248^a^	822^a^	[13]
Lwak, Kenya	October 2006- September 2009	Rural	36	Active (field clinic) + biweekly house to house visit + baseline census + health care utilization adjustment	All age; Current fever of 38 °C; OR respiratory illness^d^ OR hospitalization	25,000	77,017^e^	11,258	4185	4185	4944^e^	22	29	445^a^	[13]
Pemba, Zanzibar Tanzania	January 2010- December 2010	Rural	12	Passive (three hospital + health care utilization adjustment)	≥2mts age; Current fever of 37.5.C	500,600	53,064	3105	2209	2209	38,182	210	4	55^a^	[14]
S Asia															
New Delhi, India	November 1995-October 1996	Urban slum	12	Active (twice weekly house visit + study clinic + baseline census)	<40 years;Current fever of 38 °C for <5 years; Current fever of 38.C for ≥ 3 days for >5 years	7159	6,454^e^	1454	1217	1217	5402 ^e^	63	880	1166	[17]
Kolkata, India	November 2003-October 2004	Urban slum	12	Active (monthly household visit + 2 government hospitals + 5 study clinics + baseline census)	All age; Febrile ≥ 3 days	56,946	56,946	4378	4342	4342	56,478	122	214	216	[15]
Dhaka, Bangladesh	December 2000 -October2001	Urban slum	10	Active (weekly house visit + field clinic + baseline census)	All age; Current fever of ≥38 °C for <5 years; Current fever of ≥38.C for ≥ 3 days for >5 years	NA	12,407^e^	889	888	888	12,393^e^	49	474	395	[18]
Dhaka, Bangladesh	January2003-Januay 2004	Urban slum	12	Active (weekly household visits + field clinic + baseline census)	All age; Current fever of ≥38 °C for <5 years; Current fever of ≥38.C for ≥ 3 days for >5 years	26,586	19,710^e^	1333	961	961	14,210^e^	40	150	282	[30]
Karachi, Pakistan	June 1999-December 2001	Urban	12	Active (fortnightly households visits + two study clinics + baseline census)	<16 years of age; Febrile ≥ 3 days	41,845	41,845	7736	7415	7415	40,109	189	452	471	[19]
Karachi, Pakistan	August 2002-July 2004	Urban slum	30	Active (weekly household visit + three study clinics + motivation to private providers + baseline census)	2 to 15 years old; Febrile ≥ 3 days	11,668	29,170^e^	4198	1248	1248	8672 ^e^	49	168	565	[15]
Peri-urban Karachi, Pakistan	February 2007-May 2008	Semi-urban + Rural	15	Active (weekly household visit + local community health center + baseline census	<5 years of age; Current fever of 38 °C OR pneumococcal clinical syndrome^f^	5570	3,949^e^	3372	1165	1165	1,364^e^	16	230	1173	[21]
SE & Eastern Asia															
Hechi, Guangxi, China	August 2001-July 2002	Rural + Urban	12	Passive (5 hospitals + 23 government clinics + 99 private clinics + baseline census)	5 to 60 years of age; Febrile ≥ 3 days	97,928	97,928	1215	1215	1215	97,928	15	15	15	[15]
Jakarta, Indonesia	August 2002-July 2003	Urban slum	24	Passive (8 government public health centers + 2 government hospitals + baseline census)	All age; Febrile ≥ 3 days	160,261	160,261	6708	5775	5775	137,971	221	69	80	[15, 23]
Dong Thap Vietnam	December 1995-December 1996	Rural	12	Passive (2 health centers + 1 hospital + motivation to private providers + baseline census)	All age; Current fever of ≥38.C for ≥ 3 days	28,329	28,329	973	667	658	19,158	56	198	292	[24]
Hue, Vietnam	June 2002-June 2003	Urban	13	Passive (4 hospitals + 32 government clinics + 55 private clinics + baseline census)	5 to 18 years of age; Febrile > 3 days	84,455	84,455	3678	3611	3611	82,917	18	20	20	[15]
Summary	November 1995 to December 2010	Urban and Rural	281	Variable	Variable	4,010,372	NA	63,220	41,500	41,325	NA	1149	NA	NA	

*NA* Not available

^a^As reported by authors

^b^Denominator was corrected for dropout of eligible cases at various level of surveillance starting from health care utilization, referral to health facility, failure to collect blood sample, missing data

^c^No correction factor was available

^d^Respiratory illness was defined for children <5 years old as: cough OR difficulty breathing AND one of the following: convulsions, unable to drink fluids or unable to breastfeed, lethargic, chest in drawing, vomiting everything, stridor, oxygen saturation <90 %; and for persons ≥5 years old as cough OR difficulty breathing OR chest pain AND one of the following: temperature ≥38.0 °C and oxygen saturation <90 %

^e^Estimated in person years

^f^Pneumococcal clinical syndrome is defined by PneumoADIP investigator group; available at: Case definition for pneumococcal syndrome and other severe bacterial infections. *Clin Infect Dis.* 2009:48(suppl 2): S197-S202

When full text papers of selected studies were meticulously reviewed, we identified several reasons to argue that longitudinal studies underestimate typhoid fever incidence owing to the study design and implementation, and categorized them into six groups (Table 3). The most common problem is that surveillance sites do not capture all febrile cases because there are multiple service providers at the community such as private practitioners, traditional healers etc. who are not included in the study. We note that most passive typhoid fever surveillance studies relied upon only public health facilities.

**Table 3:** Common biases in typhoid fever surveillance and potential solutions

	Under estimation biases in typhoid fever surveillance	Potential solutions
1	All the people in the target community do not visit index facility used for surveillance	a. Conduct active surveillance by making house to house visit which is resource intensive but more precise
		b. Conduct a census and health care utilization survey, and apply a correction factor for the underutilization of health facility
2	All people visiting surveillance site and meeting inclusion criteria are not included in sampling	Estimate what proportion of people with inclusion criteria were not recruited and apply a correction factor
3	Febrile syndrome does not capture all typhoid fever infected people because some may not have symptoms severe enough to be captured and others may be asymptomatic	Broaden the inclusion criteria, particularly for younger children. This will be resource intensive.
4	Blood samples could not be collected from all eligible cases	Document blood sample collection failure along with reasons such as consent issues and apply a correction factor
5	Could not be included in data analysis for various reasons such as incomplete data, blood sample contamination	Document dropouts and apply a correction factor it
6	Blood culture does not detect all typhoid fever cases	a. Document history of antimicrobial intake prior to blood sample collection and estimate its relation to culture positivity.
		b. Apply a correction factor for blood culture sensitivity based on best applicable evidence for that settings (e.g. based on empirical research findings, literature review)

A commonly used inclusion criterion, febrile syndrome (see Table 2) is another potential source of incidence underestimation. One of the studies in Kenya used two criteria (febrile illness and respiratory illness) in urban site and three criteria (febrile illness, respiratory illness and hospitalization) in rural sites [13]. Febrile criteria had identified only 60 and 27 % of total blood culture confirmed cases reported in urban and rural sites respectively. Respiratory criteria had identified additional 38 and 50 % cases in urban and rural sites respectively. The hospitalization criteria in rural site had identified additional 23 % of cases. Total 2 % of cases were detected among persons who did not match any of the criteria in urban site. The febrile definition of fever of 38 °C for ≥3 days may limit the number of cases identified as well. The study in Karachi reported that only 24 % of febrile episode identified from house to house visits had ≥3 days of fever [19] and the rest were not referred to index surveillance facility. In some other sites only a proportion of the potential typhoid fever cases are included. Only hospitalized cases were included in Agogo study [12] and the researchers estimated that only 50 % of the cases with inclusion criteria were enrolled in the study and applied a correction factor.

Sometimes only a fraction of people identified at community reach index surveillance facility as observed in Karachi where 30 % of 4198 febrile illness cases with more than 3 days of duration presented at the health facility [19]. Even if febrile illness cases reached health facility, the blood samples couldn’t be collected from many eligible cases. In Dhaka, blood samples of only 72 % of eligible cases could be collected [30] for reasons such as parents not consenting and unavailability of logistics. Similarly, in New Delhi blood samples were collected from 84 % of eligible cases [17]. Sometimes a part of the cases are excluded from analysis because of data related or operational issues. Nearly 13 % of cases provided blood samples that were excluded in Agogo study because of incomplete data [12]. Finally, blood culture does not detect all typhoid fever cases due to lowered sensitivity related to duration of illness, amount of sample collected and problems with blood collection procedure particularly in younger children as well as prior intake of antibiotics. Of the 14 studies, eight presented information on prior intake of antibiotics [9, 10, 12, 18, 19, 21, 24, 30].

We identified potential resolutions (Table 3) for these limitations observed in each selected study, and estimated correction factors to account for under-estimation. Table 2 illustrates how denominators and numerators change when these under-estimation limitations are corrected. We present incidence rates using two methods: a) incidence based on raw surveillance data, b) surveillance methods adjusted incidence. The incidence rates are very different and amplified when corrected for surveillance limitations. The corrected incidence data was used in the estimation of global burden of typhoid fever [32].

**Fig. 3:**
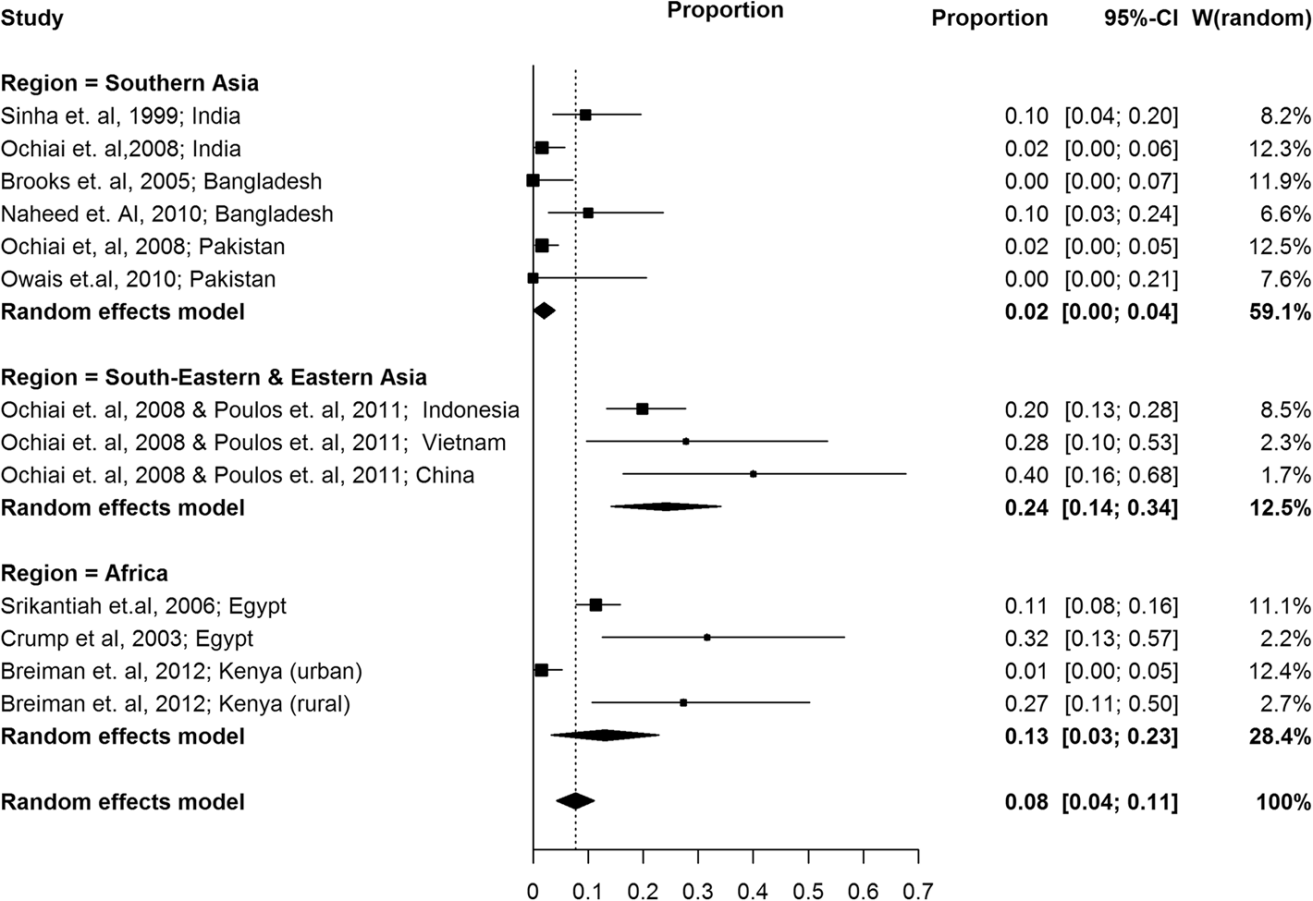
Weighted mean hospitalization rates using random effects model in selected population- based longitudinal typhoid fever studies classified by regions

We looked at the hospitalization rate in blood culture-confirmed typhoid cases from 14 selected population-based studies. The weighted mean hospitalization rates by regions using random effect models from eight studies [9, 10, 13, 15, 17, 18, 21, 30, 33] conducted in 13 sites were presented in Fig. 3. Hospitalization rate was highest in South-Eastern and Eastern Asia region which had passive surveillance. Hospitalization was least in Southern Asia where most sites had active surveillance. The active surveillance studies allow for detection of less severe cases and also likely to improve typhoid outcomes by identifying cases early. As observed in Kenya [13], hospitalization rates are likely to be higher in rural areas with poor access to healthcare, due to delays in receiving appropriate treatment. Of the 14 population-based, longitudinal studies, only one study had presented any deaths related to typhoid fever (CFR = 2.6 %; one death in 37 cases) [11].

## Discussion

In this systematic literature review we have presented prospective population-based, blood culture-confirmed typhoid fever surveillance results. In 24 years’ worth of literature, beside clinical trials, there were only 14 published studies from 15 sites. This indicates typhoid fever surveillance is not a priority despite millions of people affected. The information from some parts of the world such as South America are outdated and generated from clinical trials. The information from Africa is too minimal and comes from four countries only. There are more sites from Asia, yet its geographical representation is minimal. Large countries like India have only two sites. We show that within regions and even within countries, there is considerable variation in incidence rate estimates. One can argue that some of the variations in incidence may be attributed to strength of surveillance system that measured incidence rates. This was demonstrated at one of the surveillance sites, where involvement of private practitioners resulted in large increase in incidence rates [34].

We listed several critical points that affect the measurement of typhoid fever incidence. This included under estimation biases resulting from skewed health care utilization, limitations of eligibility criteria, sampling limitations, failure to collect blood samples, missing data and poor sensitivity of blood culture. Here we suggest how these limitations, if addressed, can improve community representativeness of surveillance (Table 3).

Surveillance sites not capturing all target population is one of the key under estimation biases that can be addressed by adding additional components to the surveillance. A baseline census followed by an active surveillance where periodic house to house visits are made to ensure all individuals with inclusion criteria could be identified and referred to index health facilities eliminates this caveat. An active surveillance allows for the identification of less clinically severe cases and may yield accurate representation of community level incidence. Such studies are often accompanied by periodic demographic census that documents migrations, births and deaths in target population. Census allows for the accurate measurement of follow-up period in person years which increases measurement precision. Based on periodic census in Dhaka study [30], it is reported that on an average individuals stayed about 10 months in 1 year surveillance. If only onetime baseline census is conducted one would overestimate the denominator calculated in person years resulting in underestimation of the incidence. But active surveillance needs dedicated staff and costs are high; likely to introduce a bias in typhoid outcomes because cases are identified quickly and treated. As periodic demographic census and active surveillance is resource intensive, having a health care utilization survey along with census is an option to lower the cost and reasonable alternative. Based on a community survey, the study from Zanzibar estimated that only 10.6 % of febrile illness cases in the target community visited the index surveillance site at Chake Chake District Hospital and applied a correction factor for the incidence [14]. Similar health care utilization survey was used in other sites as well [9, 10, 12, 13].

Surveillance inclusion criteria not capturing all typhoid fever cases is another key under-estimation bias identified. Broadening the inclusion criteria to include respiratory illness and hospitalizations in addition to febrile criteria as done in Kenya [13] is one approach to capture missing typhoid fever cases. However, these additional inclusion criteria are not specific for typhoid fever, may result in screening and testing large number of extra cases making it resource intensive. Besides enhancing the surveillance intensity, conducting health care utilization and expanding inclusion criteria, most useful measure that can limit under estimation bias is good documentation during surveillance (Table 3). It is crucial to carefully document what proportion of eligible people are a) not visited the surveillance sites, b) not included in the sampling, c) not consented for blood collection, d) could not draw blood sample, e) dropped out from the analysis. Documenting the antibiotic intake prior to blood sample collection that inhibits bacterial growth and correcting for blood culture sensitivity is another crucial factor. Until recently, most studies used a correction factor assuming 50 % blood culture sensitivity [14]. It is not sure if this number accounts factors that influence blood culture sensitivity. Documenting the history of intake of antimicrobials prior to the collection of blood samples and analyzing its implication on culture positivity is an important measure to understand blood culture sensitivity. Beyond this, it is crucial to standardize blood culture methods in multi-site studies along the side of good measures for quality control. In this regard, automated blood culture systems such as the Bactec or other commercial systems may be more reliable than studies using in-house blood culture methods.

Future studies should consider above limitations in designing the typhoid surveillance so that more precise estimation of incidence is possible. One should carefully plan surveillance to represent community either through active surveillance approaches, or setting-up of health facilities close to community for the purpose of case detection, or adjusting for health care utilization of the facility or by involving most health service providers in the defined geographical region. Dropouts at different steps of surveillance process should be documented carefully so that it can be corrected. As community based prospective surveillance is basis for global and regional typhoid fever disease burden estimates, correcting for these factors are essential [32]. The country, regional and global level disease burden estimates are powerful information for decisions on policy, financing, vaccination strategies as well as for advocacy; more precise information is valuable. Moreover, a new generation typhoid conjugate vaccine with hopes to have protection for children <5 years [3], warrants decision makers to look into new option for typhoid vaccination .

This study has limitations. Our search was confined to English language only. However, we do not think we missed many papers because a previous systematic literature review on typhoid incidence studies [35] did not find papers in Spanish, Italian, French, and Portuguese from 1980 to 2009. This review is subjected to publication biases as we did not search for unpublished information. We have presented incidence correction based on the information reported in the paper and we couldn’t correct for unreported information or factors that studies did not mention or accounted for.

## Conclusions

In conclusion, longitudinal typhoid fever studies are available only from selected geographical pockets in low and middle income countries despite its public health importance. The incidence is heterogeneous across the sites, so was the methodology of surveillance which potentially contributed to under estimation biases. Future longitudinal typhoid fever studies should consider the methodological limitation presented in this review at their study design. A precise estimation of typhoid fever incidence accounting for methodological under estimation has policy, financing and advocacy implications.

## Availability of data and materials

All the data used are presented in the manuscript.

## Additional file

Additional file 1:
**PRISMA 2009 Checklist.** (DOC 63 kb)

## Abbreviations

LMICs: low and middle income countries
PAHO: Pan American Health Organisation
WHO: World Health Organization
